# Sustainable Manufacturing of Fully Printed Zn/ZnO/CNT Schottky Diodes on Kraft Paper

**DOI:** 10.1021/acsaelm.5c02004

**Published:** 2026-01-21

**Authors:** Luís Henrique Tigre Bertoldo, Maíza Ozório, Douglas Henrique Vieira, Rogério Miranda Morais, Andrew Rollo, Jeff Kettle, Neri Alves

**Affiliations:** a Faculty of Science and Technology (FCT), Physics Department, São Paulo State University − UNESP, Presidente Prudente, São Paulo 19060-900, Brazil; b James Watt School of Engineering, University of Glasgow, Glasgow, Scotland G12 8QQ, U.K.

**Keywords:** Schottky diode, sustainable electronics, paper, printed electronic, zinc, zinc oxide, CNTs

## Abstract

The escalating generation of electronic waste underscores the critical need for sustainable alternatives to conventional electronic technologies. Printed electronics emerge as a promising approach to address this issue by incorporating sustainable materials, implementing energy-efficient fabrication methods compatible with large-area manufacturing, and integrating end-of-life (EoL) strategies to minimize the environmental impact associated with waste management. In this work, we demonstrate fully printed Schottky diodes on kraft paper substrates fabricated using zinc (Zn) as a sustainable ohmic contact, zinc oxide (ZnO) nanoparticles as the semiconductor layer, and carbon nanotubes (CNTs) as the Schottky contact. The devices were manufactured using large area deposition processes at low-temperature and with vacuum-free printing techniques. The Cheung, Norde, and Mikhelashvili methods enabled the estimation of an effective Schottky barrier height of 0.75 ± 0.04 eV, a series resistance of 2.2 ± 1.5 kΩ, and a high ideality factor of 8.0 ± 1.4, which was corrected to 5.1 when it was voltage independent. These analyses also revealed the presence of trap states and the onset of a space-charge-limited current (SCLC) regime, with these electrical properties interpreted being considered and correlated with the morphological and structural characterizations. The diode exhibited a rectification ratio of (1.6 ± 1.2) × 10^3^ and, in a proof-of-concept demonstration, successfully performed half-wave rectification, underscoring its potential for low-power and low-frequency sustainable electronic circuits on paper. Finally, life cycle assessments (LCA) showed the adopted manufacturing approaches and materials provide a lower impact route for fabricating sustainable diodes.

## Graphical abstract

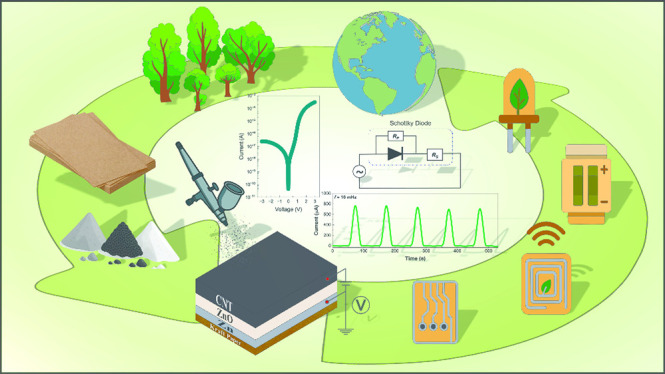

## 1 Introduction

Electronic waste (‘e-waste’) has become a major global sustainability problem with record levels of 62 million tons generated in 2022 according to the *Global E-waste Monitor 2024*,^1^ which highlights the urgent need for a step change in the design of electronics for eco-friendly disposal, reuse or recycling. The issue is a growing one and is likely to continue spreading further, due to the increasing adoption of new technologies, which will become more deeply embedded within society. Large area electronics (LAE) underpin a lot of emerging electronic areas (Internet of things (IoT), displays (including extend reality (XR), smart packaging, digitalization and Big Data) and offers an opportunity to actually reset the e-waste issue by developing electronic systems that have inherently end-of-life (EoL, cradle to cradle) solutions built in and thus do not require the same complexity of waste management. This provides an opportunity for LAE as eco-friendly devices can be manufactured using low-cost printing techniques by combining sustainable organic or inorganic materials. Unlike conventional electronics, which are designed to operate for extended periods, sustainable or ‘transient’ electronics are intended to function only for short durations.^2^ After serving their purpose, these devices partially or fully degrade into benign end-products for safe disposal. This principle has already been applied into different device systems, including biomedical,^3^ hardware security,^4^ energy harvesters,^5^ and transistors.^6,7^

One of the most critical components of a device designed for transient electronics is the substrate, because in LAE, it is the primary component of the circuit’s mass. Several sustainable materials have emerged as promising candidates in the literature, used for distinct functionalities in devices, as shown in Table 1, including polylactic acid (PLA),^8^ poly­(glycolic acid) (PGLA),^9^ poly­(vinyl alcohol) (PVA)^10^ and paper.^11^ These materials have gained increased attention due to their low embodied energy, recyclability and degradability, and reducing waste at the EoL. Different substrate materials, listed in Table 1, offer unique advantages regarding surface properties, permeability to oxygen/water, dielectric properties, as well as biodegradability. However, paper represents a practical option due to its low cost, availability, eco-friendly and renewable production process, and flexibility for scalable manufacturing processes.^11,12^

**Table 1:** Sustainable Materials for LAE

functionality in devices	sustainable and natural materials (grouped)	manufacturing techniques (examples)	ref
dielectric materials	inorganics: SiO_2_; MgO; Si_3_N_4_	conventional solution processing, wet deposition, printing, electrospinning	^22–24^
	synthetic polymers: PLA		
	naturally derived polymers: cellulose (paper), silk, shellac, gelatin, amino acids/peptides		
semiconductor materials	inorganics: ZnO; Si nanomembranes (Si NM)	solution processing, wet deposition, printing	^22–24^
	organic semiconductors: poly(3-hexylthiophene-2,5-diyl) (P3HT); diketopyrrolopyrrole (DPP)-based copolymers		
	bioderived/mixed ionic–electronic: indigo; melanin; β-carotene		
electrode/conductor/antenna materials	sustainable metals: Mg; Zn; Fe; Mo; W	metal deposition (evaporation/sputter), printing (inkjet/screen), spray/blade coating	^22–24^
	carbon-based conductors: CNTs; graphene; graphite; carbon black		
	conducting polymers: poly(3,4-ethylenedioxythiophene): polystyrenesulfonate (PEDOT:PSS); polyaniline (PANI); polypyrrole (PPy)		
substrates	naturally derived polymers: Cellulose (paper), silk, chitosan, collagen	paper processing, film casting, spin coating, electrospinning	^22,24,25^
	sustainable synthetics: PLA; PLGA; PVA; polycaprolactone (PCL)		
encapsulants and adhesives	sustainable synthetics: PLGA; PCL; PVA; poly(orthocarbonate) (POC); poly(butylene adipate-*co*-terephthalate) (PBAT)	spin/dip coating, drop casting, lamination	^24,26^
	naturally derived: starch; sucrose		

In terms of sustainable electrodes for LAE, as shown in Table 1, there are a number of options and these include carbon-based materials such as graphene, reduced graphene oxide (rGO),^13^ laser-induced graphene (LIG),^12^ carbon nanotubes (CNTs),^14,15^ and carbon based ink.^7,16^ Metallic materials are also known to degrade into benign products at the EoL and include materials such as molybdenum (Mo),^17^ iron (Fe),^17^ and zinc (Zn).^17–20^ These materials can also be printed and are typically dispersed in a solvent with a polyvinylpyrrolidone (PVP), which acts as a filler prior to deposition onto substrates. There is a limited number of eco-friendly semiconductors. ZnO provides one option as an n-type semiconductor with a wide bandgap of approximately 3.3 eV, combining functionality with sustainability. It is commonly used as the active layer in ultraviolet (UV) light and humidity sensors.^21^

Among the most practical device architectures for investigating materials and surfaces across metal/semiconductor junctions are Schottky diodes (SDs). SDs rely on a rectifying metal–semiconductor contact, with barrier height φ_b_ *=* φ_m_ *–* χ_SC_, in which the work function is (φ_m_) and the semiconductor electron affinity is (χ_SC_). Their unipolar transport supports low-voltage, and fast rectification. In addition, their simple two-terminal architecture enables the extraction of main diode parameters, such as the φ_b_, ideality factor (*n*), and series resistance (*R*_s_) from current versus voltage (*I–V*) analysis.^27,28^ This geometry also suits low-temperature processing and flexible substrates, making them practical vehicles for evaluating charge transport in sustainable device stacks.

In this context, paper substrates attach flexibility, printability, and biodegradability, aligning device studies with sustainable electronic goals.^29,30^ On paper, rectifying diodes have been demonstrated using ZnO/graphite heterojunctions for UV detection, with parameters extracted via Cheung’s method.^31^ IGZO-based Schottky barriers have also been realized on paper, including room-temperature Ru–Si–O/IGZO contacts and IGZO/Ag diodes with thermal-stability analyses.^30,32^ Other approaches include nanopaper metal/insulating/semiconductor junction (MIS) organic diodes made by inkjet printing, fully printed CNT/graphene diodes operating in the MHz range on paper, CdS diodes on free-standing cellulose, and MoS_2_/CuO piezotronics diodes.^29,33–35^ However, many reported devices still depend on metal contacts like Ag and Au, as well as on nonearth-abundant or toxic semiconductors (e.g., IGZO, In/Ga or CdS), which compromise their eco-friendliness.^29,30,32,34^ A recent advancement in greener fabrication has demonstrated fully printed diodes on paper using water-based graphene and MoS_2_ inks, thereby eliminating the need for metals and high-temperature processing.^36^ These developments highlight the importance of designing diodes in which all constituent layers are either sustainable or composed of earth-abundant materials, paving the way for sustainable and disposable electronics with reduced environmental impact.

In this study, we fabricated SDs on kraft paper, using a Zn/ZnO/CNT structure. A solution processable Zn electrode was deposited by brush coating, followed by the sequential deposition of ZnO nanoparticles (ZnO) and CNTs using spray-coating technique. For the Schottky contact, it was chosen CNTs with a work function of ∼4.95 eV, motivated by the fact that recent reports have shown promising performance of the ZnO/CNT Schottky junction.^15,37^ The morphological analysis was conducted to examine the properties of the Zn, ZnO-NPs, and CNTs thin films. Electrical characterizations provided key parameters, including rectification ratio (*RR)*, *R*_S_, *n*, and φ_b_ through Cheung’s, Norde’s, and Mikhelashvili’s methods.^38–40^ The device achieved half-wave rectification with an output current in the range of tenths of a milliampere, confirming its potential for sustainable, low-power paper electronics.

## 2 Experimental Section

### 2.1 Materials

The materials used in this study included Zn (Merck, code 96454), PVP with a molecular weight of ∼55,000 (Merck, code 856568), ethanol (Merck, code 459844), isopropyl alcohol (Merck, code W292907) Milli-Q water (resistivity of 18 MΩ·cm), ZnO nanoparticles (Merck, code 721077), CNTs (Nanoview Nanotecnologia) and kraft paper purchased from a local market.

### 2.2 Methods

Six different SDs were fabricated in a Zn/ZnO/CNT sandwich architecture on the kraft paper substrate, following the steps illustrated in Figure 1a. Initially, 2.0 g of PVP were dissolved in ethanol to obtain a 20 wt % solution, which was magnetically stirred for 1 h at room temperature. Subsequently, 15 g of Zn powder was added to this solution, and the mixture was stirred for an additional hour under the same conditions. The resulting Zn ink was deposited onto the kraft paper substrate through a shadow mask with dimensions of 30 × 5 mm, using brush coating with two layers to ensure uniform coating. The deposited films were first annealed at 100 °C to solvent evaporation, followed by further annealing at 220 °C to enhance the conductivity of the Zn electrode. An acetic acid solution (CH_3_COOH:H_2_O, 1:5 ratio) was sprayed onto the Zn surface in order to remove the native oxide layer and promote the coalescence of a continuous conductive Zn track.^17,18^

**Figure 1:**
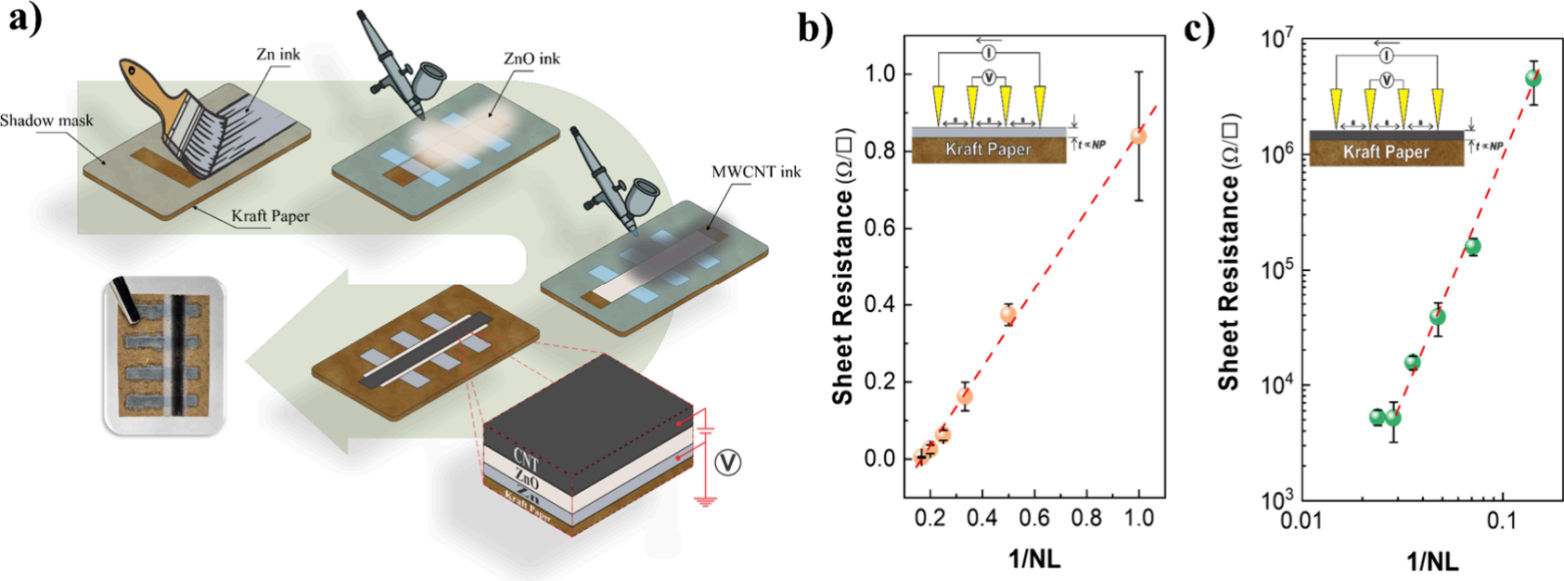
(a) Step-by-step fabrication of the SD, including a cross-sectional schematic and a photograph of the Zn/ZnO/CNT device on kraft paper. Sheet resistance as a function of the inverse number of printed layers (1/NL) for (b) Zn and (c) CNTs. Inset: schematic of the four-point probe measurement.

The ZnO solution was prepared by dispersing ZnO nanoparticles in a 1:1 mixture of Milli-Q water and isopropyl alcohol to achieve a ZnO concentration of 10% wt. Next, 10 mL of the solution was deposited onto the Zn electrode by spray-coating, with the substrate preheated to 120 °C. To complete the SD fabrication, the CNTs ink was dispersed 1:1 in isopropyl alcohol and deposited by spray coating at 120 °C through a shadow mask. Figure 1a also presents a cross-sectional illustration and a photograph of the completed diode. In addition to the SDs, Zn and CNTs samples were also fabricated on paper substrates for four-point probe measurements.

### 2.3 Characterization

Scanning Electron Microscopy (SEM) images of the films surface morphology were acquired using a Carl Zeiss EVO LS 15. The SEM images were analyzed with ImageJ software to determine the average particle diameter. Structural characterization was carried out by X-ray Diffraction (XRD) using a Shimadzu XRD 6000. Profilometry measurements were performed using the Bruker DektakXT to obtain the films thickness and roughness. Four-point probe measurements of the CNTs film and Zn electrode were performed with an Ossila T2001A4 to obtain sheet resistance (*R*_□_). *I–V* characteristics of the SDs was measured with a Keithley SCS-4200 semiconductor characterization system. For the half-wave rectification test, a Rigol DG822 Pro waveform generator was coupled to the Keithley SCS-4200.

## 3 Results and Discussion

### 3.1 Electrical Characterization of Zn and CNT Tracks

Printed conductors on paper substrates are highly susceptible to surface roughness (8.8 ± 3.6 μm for the kraft paper used in this work) and intrinsic porosity, which facilitate ink infiltration into the paper bulk.^41,42^ Consequently, achieving a continuous, percolating film with reliable electrical performance depends strongly on the thickness of the deposited conductive layer. To investigate this effect, four-point probe measurements were carried out on Zn and CNTs electrodes printed on kraft paper to determine the *R*_□_ as a function of the number of printed layers (e.g., increasing thickness), as shown in Figure 1b and Figure 1c, respectively. Zn tracks are particularly prone to rapid oxidation under ambient conditions, which increases *R*_*□*_ and remains a key challenge for Zn-based pastes and inks.^18^ The *R*_□_ of the Zn electrodes for two layers were measured at an average 370 ± 3 mΩ/□ (Figure 1b).

Since *R*_*□*_ *=* ρ/*t*, a linear relationship is expected between the sheet resistance and the reciprocal of the number of layers (1/NL), which is in good agreement with the Zn data and indicates a uniform stacking of layers. However, even for very high thickness (*t* → ∞), although *R*_*□*_ would ideally approach 0 Ω, this is not observed. The extrapolation leads to a negative value, which has no physical meaning. Two effects may contribute to the fact that the extrapolation does not reach zero. The first is the presence of oxidized surfaces between metallic grains, which introduces a series resistance. In this case, the extrapolation for 1/NL → ∞ would lead to positive values of sheet resistance, which is not observed.^43^ The second effect arises from the high roughness of the paper substrate, which requires the deposition of several layers before percolation of the metallic film starts. This effect explains why the extrapolation actually results in negative values. Therefore, the number of layers that deposited the material required to initiate percolation is defined as critical number (*N*_c_), which can be obtained from the intercept of the linear fit on the *x*-axis and, in this case, is approximately 8 layers.^44^

The CNT curve showed an exponential behavior in linear scale (see Figure S1 in Supporting Information), so the curve was plotted in log–log to compare with the Zn curve (Figure 1b), as shown in Figure 1c. Unlike the Zn film, the CNT thin films behave as a percolating, junction-limited network rather than a uniform conductor.^45^ The sheet resistance exhibits a power-law dependence on coverage, resulting in a linear behavior on log–log scales, where an average *R*_*□*_ of 5.2 kΩ/□ is exhibited with 35 spray-coated layers. Similarly to Zn, even at higher thickness the CNT electrode *R*_*□*_ does not reach 0 Ω, because CNT–CNT junctions introduce a finite series resistance.^45^ But this effect would lead to the extrapolation to a positive value at zero. A closer examination shows that this trend is not verified; instead, in this case, there is a tendency to cross the *x*-axis at a value greater than zero, resulting in a negative sheet resistance when extrapolated to 1/NL → ∞. Thus, is because the effect of paper roughness is also present here, requiring *N*_c_ depositions for percolation to occur. For CNTs, this *N*_c_ is approximately 60 layers.

### 3.2 Morphological Analysis of Zn, ZnO, and CNTs

Following the electrical characterization of electrodes, the surface morphology of the Zn, CNTs, and ZnO thin films were investigated by SEM images. As shown in Figure 2a, the surface of the Zn electrode after acetic acid treatment exhibits spherical shaped Zn particles. Zareei et al. and Feng et al. reported similar morphological structures for Zn electrodes in their studies, with irregular, well-defined acetic acid sintered spherical Zn particles interconnected by PVP matrix.^17,18^ The average particle diameter was estimated as 1.8 ± 1.1 μm, and the Zn thickness for two layers was 39.5 ± 9 μm. Despite the differences recorded in Zn electrical properties, no significant microstructure variations were verified when compared with the untreated Zn electrodes. Figure 2b,c presents the XRD patterns of Zn samples before and after undergoing thermal and acetic acid treatments, aimed at assessing potential structural modifications. Overall, no significant changes in the crystal structure were observed. However, an increase in the main Zn peaks (002 and 101) was detected. When acetic acid is sprayed onto the oxidized Zn surface, it reacts with ZnO according to the following reaction:^18^

$$\mathrm{ZnO}+2{\mathrm{CH}}_{3}\mathrm{COOH}\to \mathrm{Zn}{\left({\mathrm{CH}}_{3}\mathrm{COO}\right)}_{2}+H_{2}O$$ 1

**Figure 2:**
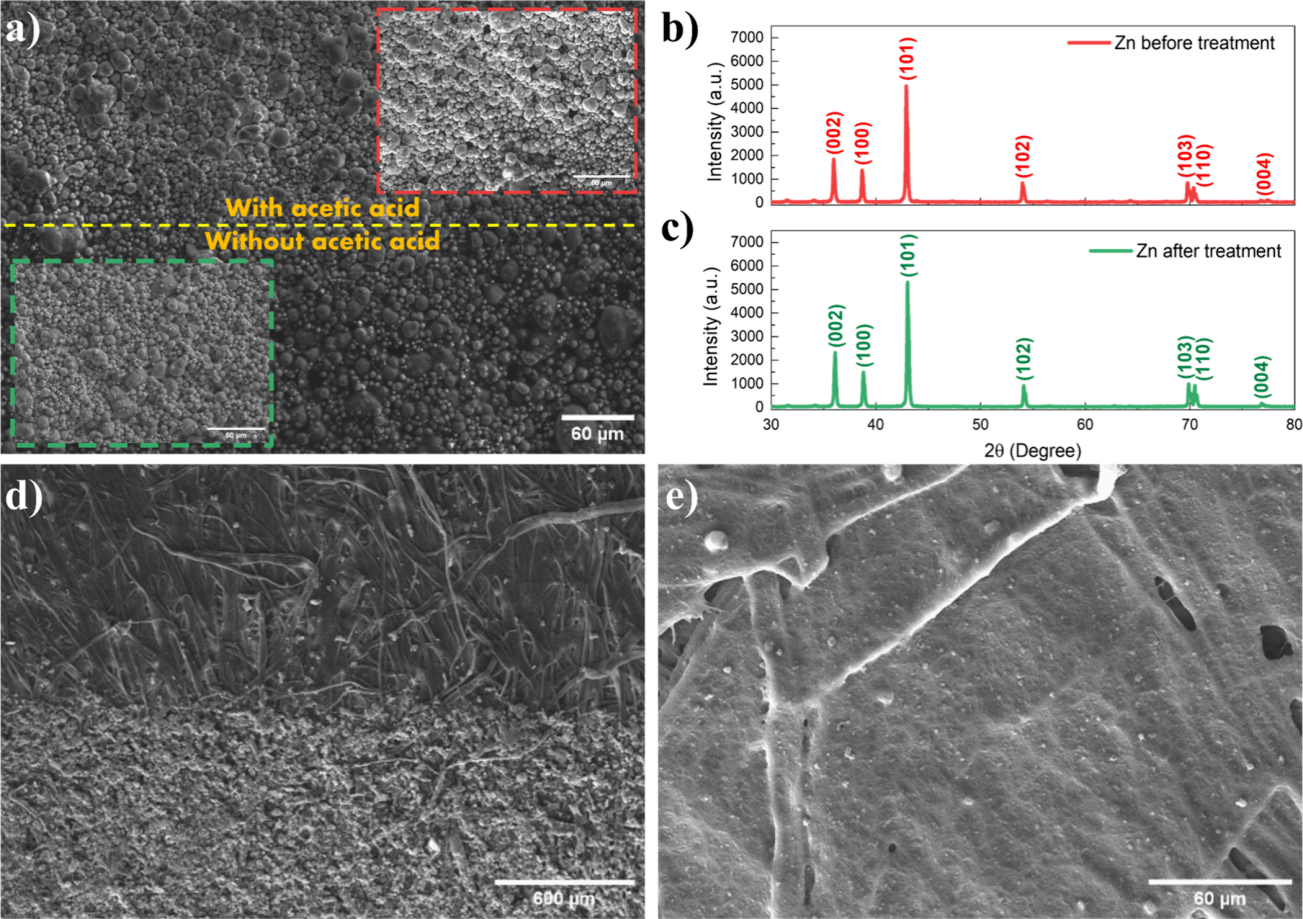
(a) SEM images of the Zn surface with and without acetic acid treatment and annealing. XRD patterns of Zn:^46^ (b) before heat and acetic acid treatment and (c) after heat and acetic acid treatment. (d) ZnO-NP film interface with the kraft paper substrate. (e) CNT film on kraft paper at 100× magnification.

During the subsequent heating at 220 °C, Zn­(CH_3_COO)_2_ decomposes and the water evaporates. As a result, the removal of ZnO and acetate species exposes more metallic Zn, which explains the increase in the main Zn diffraction peaks.

Figure 2d presents the SEM image of the boundary region between the ZnO-NPs film and the kraft paper substrate. The fibrous structure of the cellulose is clearly visible, and the ZnO film coats the fibers with good physical adhesion while preserving their morphology. Such topographical conformity is good for mechanical flexibility of the device. However, the inherent roughness of the paper may introduce artifacts that could impact the semiconductor electrical performance. Figure 2e shows the CNTs thin film deposited onto the kraft paper fibers at 100× of magnification, also conformally covering the fibers while maintaining their morphology. Figure S2 shows a cross-sectional SEM image of the Zn/ZnO interface, revealing the formation of a continuous ZnO layer on top of the metallic Zn bottom electrode (2500× magnification). The image indicates good adhesion between the oxide layer and the metal, with the ZnO film displaying a thickness of approximately 6.4 μm.

### 3.3 Schottky Diode Characterization

Electrical characterizations of the SDs were carried out on six different devices (Figure S3 in Supporting Information), so the reported figures-of-merit (FoMs) correspond to the average values across these devices. There is a clear asymmetry in the *I–V* characteristic curve shown in Figure 3a, evidencing the typical rectifying behavior of an *n*-type SD, with a turn-on voltage (*V*_ON_) of 1.7 ± 0.2 V. This rectification originates from potential barrier at CNT/ZnO interface, with the expected Schottky barrier height φ_b_ *=* φ_*m*_ *–* χ_SC_ estimated to be approximately 0.78 eV, calculated based on the work function of CNTs (φ_CNTs_ *=* 4.95 eV)^47^ and the χ_ZnO_ of ZnO (χ_ZnO_ *=* 4.17 eV),^27^ as depicted in the energy band diagram of the CNT/ZnO junction in Figure 3b. To better highlight the magnitude of the rectification, Figure 3a also presents the *I–V* plotted in a *y*-axis semilogarithmic scale, where there is a *RR* of (1.6 ± 1.2) × 10^3^ over a voltage range from −3 to 3 V. Under reverse bias, the saturation current (*I*_s_) is relatively high, indicating the presence of a parallel conduction path across the junction barrier, while in forward bias the current reaches approximately 0.1 mA, limited by the semiconductor bulk. In terms of an equivalent circuit, these nonidealities can be represented by a shunt resistance (*R*_sh_) associated with the leakage in reverse bias, and a series resistance (*R*_s_) associated with current transport in forward bias.

**Figure 3:**
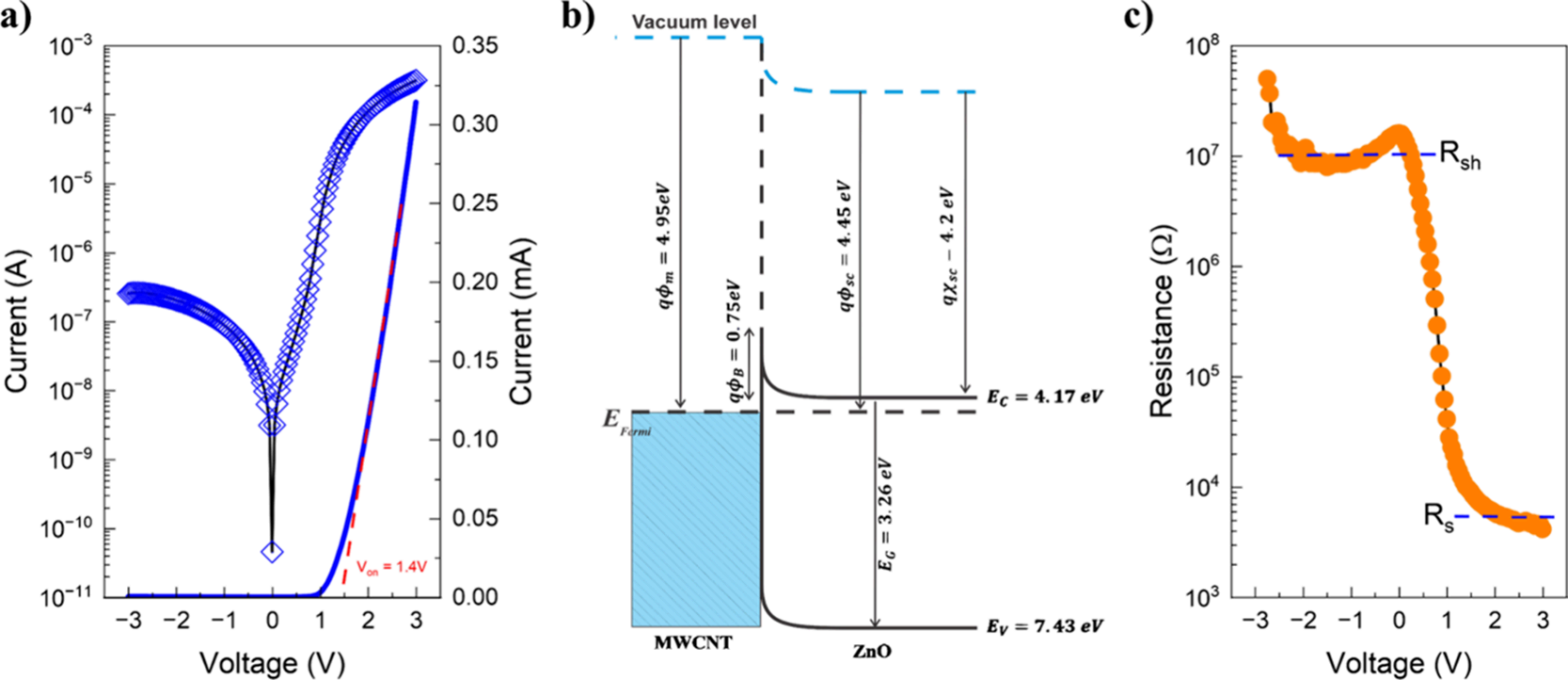
Characteristic curves for the Zn/ZnO/CNT SD: (a) linear and semilog *I–V* curves; (b) zero bias CNT/ZnO energy band diagram; (c) *R*_i_ *–V*.

Under reverse bias, the saturation current (*I*_s_) is relatively high, indicating the presence of a parallel conduction path across the junction barrier. In contrast, under forward bias, the current reaches approximately 0.1 mA, limited by the semiconductor bulk. In terms of an equivalent circuit, these nonidealities can be modeled by a shunt resistance (*R*_sh_), associated with leakage in reverse bias, and a series resistance (*R*_s_), related to current transport in forward bias. Since the semiconductor layer consists of ZnO nanoparticles, the presence of interparticle voids, grain boundaries, and bulk defects is expected, which can contribute to additional parallel leakage pathways within the film and, consequently, decrease the effective *R*_sh_.

A relatively high *I*_s_ also suggests a nonideal metal/semiconductor interface, typically attributed to a barrier inhomogeneities that facilitate additional leakage paths through thermionic-field emission or trap-assisted tunneling. Similar relationships between elevated reverse current and poor interface quality have been reported in Schottky devices with engineered interfaces, such as Ni/HfO_2_/β-Ga_2_O_3_ and metal/polymer/semiconductor structures, where improved interface passivation leads to higher effective barrier heights and significantly reduced leakage currents.^48,49^

According to the proposed circuit, the total current of the diode can be described by the following expression:^50^

$$I=I_{s}\left[\exp\left(\frac{q(V-IR_{s})}{nkT}\right)-1\right]+\frac{V-IR_{s}}{R_{\mathrm{sh}}}$$ 2

where *n* ≥ 1 is the ideality factor, *k* is the Boltzmann constant, and *T* is the ambient temperature in Kelvin. This equation shows that, by calculating the differential resistance (*R*_i_ *=* d*V/*d*I*) it is possible to estimate both *R*_s_ and *R*_sh_. Figure 3c shows the *R*_i_ *–V* curve where a plateau under reverse bias yields *R*_sh_ *=* 10^7^ Ω, and under forward bias, a low resistance value is observed, with *R*_*s*_ *≈* 4.7 kΩ. Given the inherent defects associated with printing deposition processes and the substrate roughness, we regard these parameters as suitable within the current state-of-art for fully printed SD on paper substrates.

To further probe the diode properties, such as the effective φ_*b*_ and the *n*, the analytical methods proposed by Cheung^38^ and Norde and Bohlin^39,51^ were applied. Cheung’s method relies on two distinct analyses performed in the *I–V* SD curve. First, the d*V*/dln­(*I*)*−I* plot, which follows the expression

$$\frac{dV}{d\ln(I)}=IR_{s}+\frac{nkT}{q}$$ 3

where *q* is the electron charge. The slope of this linear relation provides the *R*_s_, while the linear coefficient yields *n*. The calculated values of *n* and *R*_s_ are shown in Table 2 (Cheung – d*V*/dln *I*). Second, the *H*(*I*) versus *I* plot, given by

$$H(I)=V-\frac{nkT}{q}\ln\left(\frac{I}{AA^{*}T^{2}}\right)=IR_{s}+n\varphi_{b}$$ 4

where *A* is the effective diode area, corresponding to the semiconductor region between the electrodes, here *A ≈* 4.4 × 10^–2^ cm^2^, and *A* ^*^ is the Richardson constant, which is 36 Acm^–2^K^–2^ for ZnO.^21^ Again, the slope provides *R*_s_ while the intercept yields φ_b_. The estimated φ_b_ and *R*_s_ are also presented in Table 2 (Cheung – *H­(I)*). Figure 4a shows both the d*V*/dln­(*I*) and *H*(*I*) versus *I* plots.

**Table 2:** Calculated SD Parameters Obtained Using Cheung’s and Norde’s Analytical Methods with Corresponding Errors Derived from Six Measured Devices

method	*n*	φ_*b*_ (eV)	*R*_s_ (kΩ)
Cheung – d*V*/dln *I*	8.0 ± 1.4		2.2 ± 1.5
Cheung – *H*(*I*)		0.75 ± 0.04	2.3 ± 1.5
Norde		0.74 ± 0.04	3.3 ± 3.2

**Figure 4:**
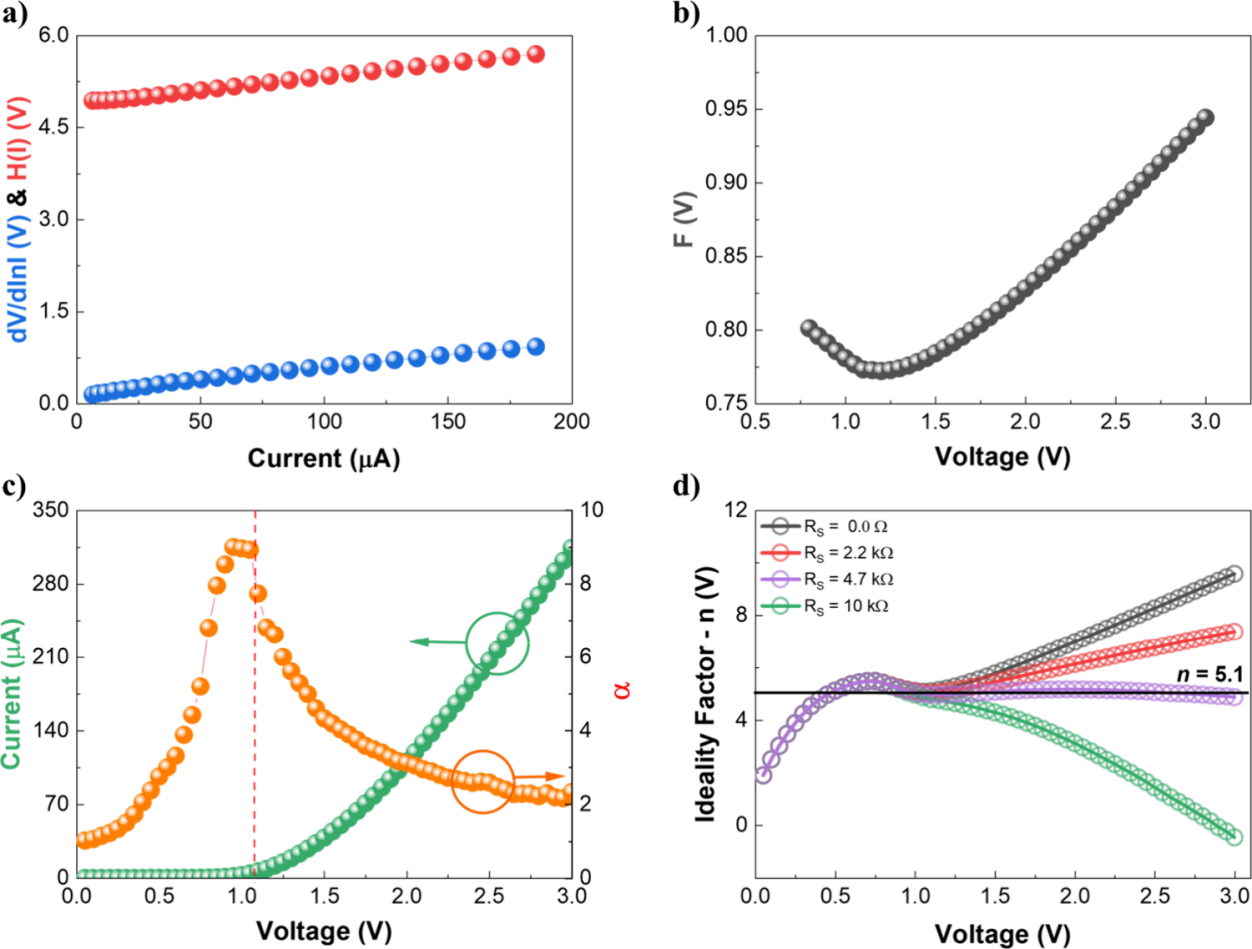
Characteristic curves obtained using the analytical method of (a) Cheung and (b) Norde. (c) Differential slope α–*V* plot contrasted with the linear scale *I–V* curve. (d) *n*(*V*)*−V* curve obtained using the Mikhelashvili method.

Norde’s method consists in analyzing the minimum point of the *F*(*V*,α) versus *V* plot, in which

$$F\left(V,\alpha \right)=\frac{V}{\alpha }-\frac{kT}{q}\ln\left(\frac{I}{AA^{*}T^{2}}\right)$$ 5

being α the first integer greater than *n*. Figure 4b shows the *F*(*V*,α) curve, from which the voltage at minimum (*V*_0_) and the corresponding *F*(*V*_0_,α) are used to calculate φ_b_ and *R*_s_ according to the following equations

$$\varphi_{b}=F(V_{0},\alpha )+\left(\frac{\alpha -n}{n}\right)\left(\frac{V_{0}}{\alpha }-\frac{kT}{q}\right)$$ 6

$$R_{s}=\frac{kT}{q}\left(\frac{\alpha -n}{I_{0}}\right)$$ 7

where *I*_0_ is the current *V*_0_. The parameters calculated using Norde’s method are also reported in Table 2 (see Table S1 in Supporting Information for each device’s value).

The high value for *n* observed for the SDs is primarily attributed to the nonuniformity of the ZnO-NPs films, particularly in regions near the Ohmic contact. Surface porosity, nonpercolative regions, and high roughness, as evident in the SEM images (see Figure 2), suggest possible tunneling pathways that deviate the device from the ideal thermionic emission model (which would yield *n* = 1). Consequently, charge transport is likely dominated by alternative mechanisms, such as trap-assisted conduction, tunneling, or space-charge-limited current (SCLC). Furthermore, natural oxidation of Zn and poor contact conformity may exacerbate interfacial recombination, thereby further degrading device performance over time.^27,28,52,53^ The kraft-paper Zn/ZnO/CNT SD exhibits a higher ideality factor (*n* ≈ 8) than the Al/ZnO/CNT SD fabricated on a glass substrate, as reported in a previous study^15^ (*n* ≈ 2), indicating a stronger influence of nonideal transport mechanisms in the paper-based device. Nevertheless, despite the rough and porous nature of the paper substrate and the use of a Zn electrode, the present device delivers a forward current comparable to that obtained in the glass-based counterpart. This comparison indicates that paper substrates enable the fabrication of operational SDs with electrical characteristics comparable to those of glass-based devices, despite the influence of a higher ideality factor.

The estimated Schottky barrier height of 0.75 ± 0.04 eV is close to the expected theoretical value of 0.78 eV. Although consistent values were obtained, they may present voltage-dependence due to the presence of lateral inhomogeneities in the barrier^54^ from contributing factors such as impurities at the CNT/ZnO interface, electronic fluctuations, trap states within ZnO bandgap, nonuniform thickness, voltage drops across interfacial layers, or even direct tunneling paths between the electrodes.^21^ These artifacts may originate from morphological issues intrinsic to printed device fabrication, ultimately leading to an underestimation of the diode parameters when extracted using conventional analytical methods.^55^

The artifacts, related to interface states and others, can cause both the *n* and the φ_b_ to exhibit voltage dependence, denoted as *n*(*V*) and φ_b_(*V*). While Cheung’s and Norde’s methods provide reliable parameter extraction under the assumption of a nearly constant barrier, the Mikhelashvili method,^40^ is particularly useful as it accounts for the voltage-dependent behavior of the diode. Following Mikhelashvili’s method, the differential slope (α = d­(ln *I*)/dln­(*V*)) versus *V* plot is presented in Figure 4c. At zero bias *I* ∝ *V* ^1^ indicates ohmic injection. As the applied voltage increases, α rises, indicating the progressive filling of trap states. An α peak is observed at approximately 0.95 V, where α ≈ 9, followed by a gradual decrease with increasing voltage due to the *IR*_s_ drop, until it reaches α = 2, which is characteristic of trap-free SCLC, being consistent with previous reports.^15^ For better visualization, the *I–V* curve was plotted alongside the α–*V* curve in Figure 4c. It can be observed that the effect of *IR*_*s*_ begins to prevail at voltages greater than 1 V (orange curve in Figure 4c), a region where an increase in current starts to be observed.

Figure S4 displays the log–log current versus voltage characteristics of the Zn/ZnO/CNT SD. This plot confirms the formation of distinct power-law regimes, in which the current scales with voltage as *I* ∝ *V* ^α^. Particularly, at low voltages there are a close to unity α, indicative of an initial ohmic response. As the voltage increases, the slope approaches α ≈ 2 which is characteristic of shallow-trap SCLC. This regime is followed by a trap-filling-limited current (TFLC) region, where α ≫ 2, and finally by a trap-free SCLC regime once the traps are fully filled. Such a sequence of transitions is typically observed in Schottky diodes.^56^ From the trap-filling limit voltage (*V*_TFL_), identified as the bias marking the transition from TFLC to trap-free SCLC, the trap density (*N*_t_) can be estimated using^57^

$$V_{\mathrm{TFL}}=\frac{qN_{t}t^{2}}{2\varepsilon \varepsilon_{0}}$$ 8

where *q* is the elementary charge, ε_0_ is the vacuum permittivity, ε is the relative permittivity of the semiconductor, and *t* is the active layer thickness. Using the film thickness obtained from Figure S2 and the *V*_TFL_ = 1.7 V obtained from Figure S4, a trap density of *N*_t_ = 3.9 × 10^13^ cm^–3^ is estimated. This value is consistent with reported trap densities for ZnO nanostructure-based devices, such as ZnO nanowires with *N*_t_ ∼ 5.76 × 10^13^ cm^–3^ reported by Guruprasad et al.^58^

Using eq 9, the *n*(*V*)*−V* characteristics were plotted for the series resistance values obtained via the Cheung/Norde methods (*R*_s_ = 2.2 kΩ), the differential resistance method (*R*_s_ = 4.7 kΩ), the ideal case (*R*_s_ = 0), and one additional arbitrarily higher resistance for comparison (*R*_s_ = 10 kΩ), as shown in Figure 4d. The curves exhibited spreading above 1 V, with ideality factor *n* becoming voltage-dependent for most cases. When using the *R*_s_ value obtained via the differential resistance method, *n* remains nearly constant at 5.1 (horizontal black line), indicating that this value best approximates the actual voltage-independent ideality factor. In contrast, the *n* obtained via the Cheung method is overestimated due to the aforementioned artifacts, while the arbitrarily higher resistance leads to underestimated low *n*(*V*). These findings highlight the need for complementary evaluation methods are necessary. Such methods help probe and mitigate possible artifacts arising from the specific boundary conditions of each method.

$$n=\frac{qV}{kT}\left(1-\frac{IR_{s}}{V}\right)\left(\frac{1}{\int_{V_{0}=3kT/q}^{V}\frac{\alpha }{V}dV}\right)$$ 9

In the literature, there have been reported only a few studies on the use of Zn as electrode in diodes.^59–62^ Salari et al.^59^ demonstrated a SD based on a Zn/ZnO/Si/Au structure, which exhibited excellent performance, with a *RR* greater than 10^4^, a greater than unity *n*, and φ_b_ exceeding 0.8 eV. However, the entire fabrication process requires vacuum conditions, and the use of Si/Au electrode increases the environmental impact of the device. Jin et al.^62^ reported a heterojunction diode with a Zn/ZnO/PEDOT:PSS/Ag architecture, fabricated on a Zn wafer. While the ZnO and PEDOT:PSS layers were deposited under ambient conditions, the Ag top electrode was thermally evaporated, with Ag known to be environmentally toxic, especially in aquatic ecosystems.

The performance of the ZnO-based SDs fabricated here was benchmarked against heterojunction and Schottky diodes reported over the past six years, as summarized in Table 3. The key diode metrics, *RR*, *R*_s_, and *n*, extracted via Cheung’s method, are consistent and, in several cases, comparable to literature values; among all the parameters, *RR* stands out at (1.6 ± 1.2) × 10^3^. Unlike most devices in Table 3, which use conventional, nonsustainable substrates (Si, glass, PET) that typically account for >90% of a device’s mass, and rely on vacuum-based processing, the SD manufactured in this work is a combination of competitive electrical performance, low-energy fabrication via printing techniques (brush and spray coating techniques) that are compatible with roll-to-roll scale-up (e.g., slot-die, gravure, flexographic), and all components of the device (paper substrate, ZnO layer, and CNT electrodes) are fully sustainable. This balance of performance, scalable processing, and sustainability demonstrates the strong potential of this SD as a sustainable alternative for next-generation electronic applications, capable of delivering performance while minimizing environmental impact at the EoL.

**Table 3:** Literature Comparison of ZnO Diode Parameters Obtained Using Cheung’s Method

substrate	diode type	ZnO deposition	*RR*	*R*_s_ (kΩ)	*n*	ref/year
PET	Schottky	spray-coating	10	0.66	5.6	^63^/2020
glass	Schottky	spray-coating	10^4^	6.2	2.2	^15^/2022
Si-wafer	heterojunction	thermal evaporation		4	10	^64^/2022
Si-wafer	heterojunction	spin coating	50	0.19	4.2	^65^/2023
Si-wafer	heterojunction	spin coating	30	0.42	7.7	^66^/2023
Si-wafer	heterojunction	drop cast	47	3	4.25	^67^/2024
Si-wafer	heterojunction	spin coating	1.3 × 10^4^	3	10.7	^68^/2024
kraft paper	Schottky	spray-coating	(1.6 ± 1.2) × 10^3^	2.2 ± 1.5	8 ± 1.43	this work

ZnO nanoparticle dispersion was maintained at 10 wt % in a water/isopropyl alcohol, a condition that resulted in a continuous semiconductor layer. Although a systematic variation of ZnO concentration was not performed here, changes in concentration are expected to have a strong impact on film thickness and morphology and, consequently, on SD performance. Lower concentrations would produce thinner and less percolative ZnO layers, increasing the series resistance and the likelihood of shunt pathways across the film or local Zn–CNT shorts, thereby reducing the rectification ratio and intensifying barrier inhomogeneities. Conversely, higher concentrations would tend to improve film continuity, enhance the effective electron mobility (μ_eff_),^69^ and suppress leakage current.

### 3.4 Half-Wave Rectification Properties

To evaluate the applicability of this sustainable paper-based SD architecture in electronic circuits, we performed a proof-of-concept measurement of half-wave rectification. The equivalent circuit of the SD under AC measurement is shown in Figure 5a. In this circuit, the series resistance *R*_s_, being relatively high, effectively acts as the load resistance when the device is subjected to an alternating voltage. Figure 5b shows the sinusoidal input voltage, with an amplitude ranging from −3 to 3 V at a frequency of 10 mHz, which is expected to produce a half-wave current output, allowing the positive current to pass. Figure 5c presents the measured current versus time (*I–t*) response of the Zn/ZnO/CNT SD, demonstrating the half-wave rectification proof-of-concept. The device exhibits the expected rectification behavior over five consecutive cycles and demonstrates bias stability. These results demonstrate the device’s potential suitability for integration into low-frequency electronic circuits, supporting the development of sustainable and fully printed electronics on paper substrates.

**Figure 5:**
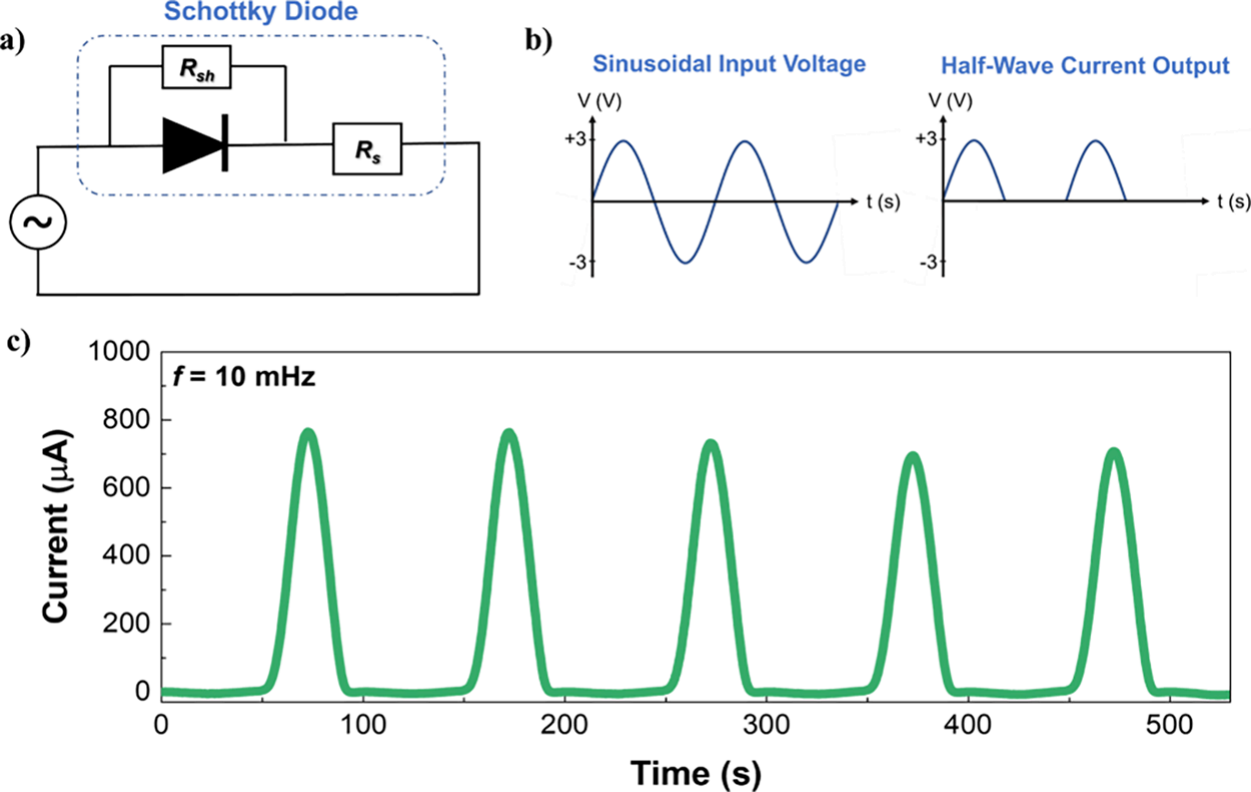
(a) Equivalent circuit of the SD under AC measurement. (b) Applied waveform and expected half-wave rectification response. (c) Current versus time curve under a sinusoidal voltage sweep from −3 to +3 V at *f* = 10 mHz.

### 3.5 Life Cycle Comparison

Figure 6a compares the environmental impacts of the sustainable diode discussed in this work (highlighted in green), produced under research-scale conditions, with those of a commercially manufactured signal diode (DO-214/219, shown in black), across multiple categories using the *Centrum voor Milieukunde Leiden* (CML) 2016 framework. The assessment used the CML impact categories, including Acidification Potential (AP), Abiotic Depletion Potential (ADP), Eutrophication Potential (EP), Freshwater Aquatic Ecotoxicity Potential (FAETP), Global Warming Potential (GWP), Human Toxicity Potential (HTP), Ozone Depletion Potential (ODP), Photochemical Ozone Creation Potential (POCP), and Terrestrial Ecotoxicity Potential (TETP), with data sourced from the Ecoinvent database. Each category represents a different indicator and uses a single diode as the comparative unit. In almost every category, the sustainable diode achieves an equal or lower impact than the commercial reference. This improvement stems from the use of Zn, zinc oxide, CNTs, and kraft paper as the substrate, compared to the refined metals, silicon processing, critical raw materials (CRMs), and epoxy compounds used in the signal diode. Overall, the sustainable diode achieves a reduced environmental impact compared to the signal diode, highlighting the importance of material substitution to achieve more sustainable discrete devices. Even though the sustainable diode is manufactured at laboratory scale, inflating per-unit electricity use, it still delivers better environmental performance. Figure 6b details the footprint by material and process stage, showing that most impacts arise from ink preparation, particularly solvent-based emissions and materials processing. This indicates that material formulation and design optimization are the main levers. Refining processes to reduce electricity and solvent use can further mitigate impacts.

**Figure 6:**
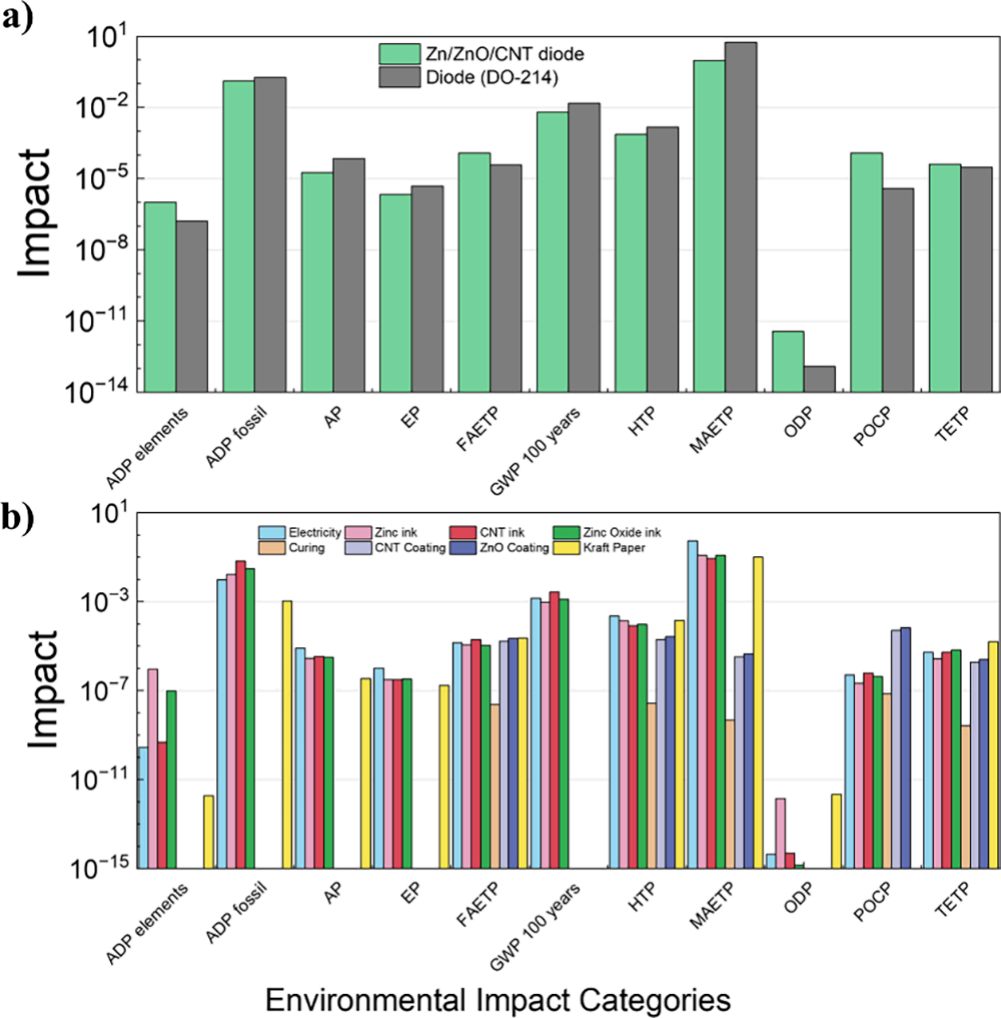
(a) Environmental impacts of commercial (gray) and sustainable (green) diodes using the CML 2016 method, showing lower impacts for the sustainable device across multiple categories; (b) breakdown of a single sustainable diode showing the processing stages.

## 4 Conclusions

In conclusion, this work demonstrates the successful fabrication of sustainable, conductive Zn and CNT tracks on kraft paper, whose electrical properties are strongly governed by the interplay between substrate morphology and film thickness. Surface treatment of the Zn electrodes improved conductivity, yielding a *R*_*□*_ of 370 ± 3 mΩ/□ with two brush-coated layers. Fully printed Schottky diodes were then fabricated using Zn and CNT electrodes with ZnO as the semiconductor, all components are sustainable and solution-processed at low temperatures, providing a lower-impact alternative to conventional diodes. The device exhibited *RR* of (1.6 ± 1.2) × 10^3^, φ_b_ of 0.75 ± 0.04 eV, and a *R*_s_ of 2.2 ± 1.5 kΩ, values comparable to reports for devices made with conventional materials and techniques. The Mikhelashvili method proved particularly effective for determining the ideality factor, allowing to obtain a voltage-independent *n* = 5.1. Electrical analysis revealed trap effects as a function of bias, with a transition from ohmic injection to trap-filling and ultimately trap-free SCLC regimes as the voltage increased. Practical operation was confirmed via half-wave rectification, indicating feasibility for low-power, low-voltage circuits. A LCA comparing the sustainable and commercial diodes highlights lower or comparable impacts for the sustainable device across categories. Analysis of a single sustainable diode attributes this chiefly to avoiding CRMs and epoxy compounds. Collectively, these results show that replacing conventional materials with zinc, zinc oxide, and carbon nanotubes on paper offers a clear route to reducing the environmental footprint of electronics. The platform established here provides a foundation for future research in sustainable electronics, with promising pathways toward eco-friendly tracks and devices for disposable sensors, transient electronics, and large-area electronics.

## Supplementary Material



## Notes

Sheet resistance as a function of 1/NL plotted on linear and semilogarithmic scales for CNT thin films deposited by spray coating; SEM cross-sectional image of the Zn/ZnO layers highlighting the ZnO film thickness; *I–V* characteristic curves of the six Zn/ZnO/CNT SD samples; table summarizing the main SD parameters extracted from the six devices; log–log plot of the *I–V* curve showing that different linear fits in different voltage regions yield different α slopes (PDF)
