# Supplementary material for: Sustainable Manufacturing of Fully Printed Zn/ZnO/CNT Schottky Diodes on Kraft Paper
Source: ACS Appl Electron Mater. 2026 Jan 21;8(3):1088–98. doi: 10.1021/acsaelm.5c02004 (PMC12895407; doi:10.1021/acsaelm.5c02004)
Supplement: Supplementary file 1 [file el5c02004_si_001.pdf]

Supporting Information

Sustainable Manufacturing of Fully Printed Zn/ZnO/CNT Schottky Diodes on Kraft Paper

Luís Henrique Tigre Bertoldo[a], Maíza Ozório[a], Douglas Henrique Vieira[a], Rogério Miranda Morais[a], Andrew Rollo[b], Jeff Kettle[b], and Neri Alves[a]*

[a] São Paulo State University – UNESP, Faculty of Science and Technology (FCT), Physics Department, Presidente Prudente, São Paulo 19060-900, Brazil.

[b] University of Glasgow, James Watt School of Engineering, Glasgow, Scotland G12 8QQ, UK.

*e-mail: luis.bertoldo@unesp.br

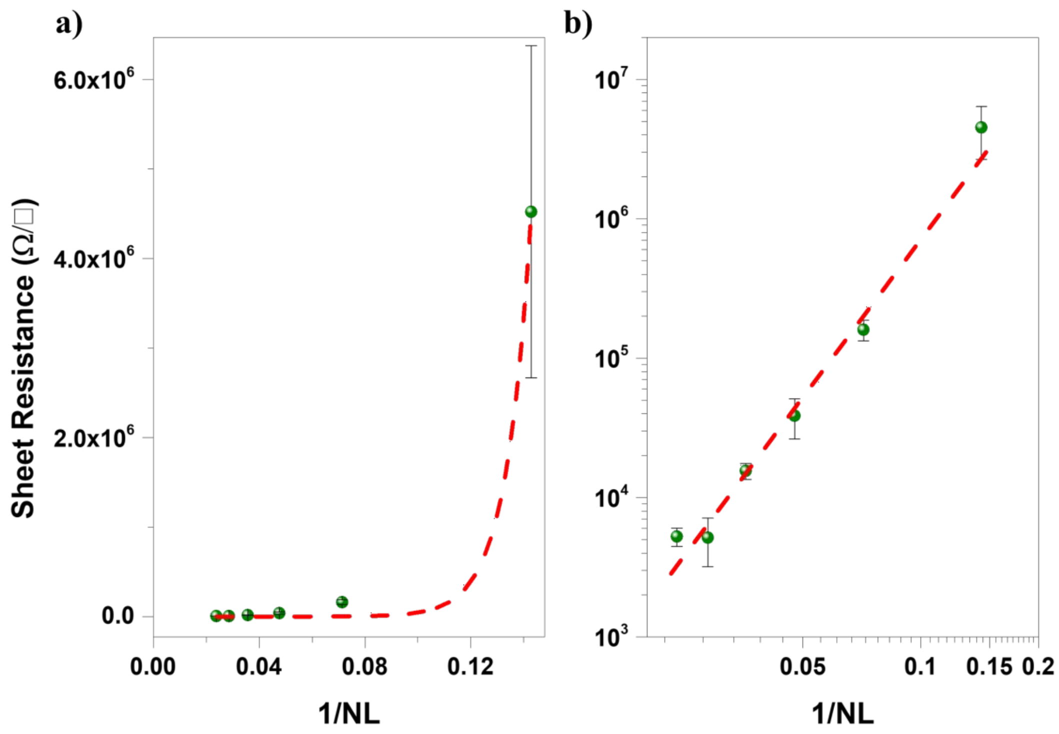

Figure S1. Sheet resistance as a function of the inverse of the number of printed layers (1/NL) for CNT thin films on kraft paper: **a)** linear scale; **b)** log–log scale.

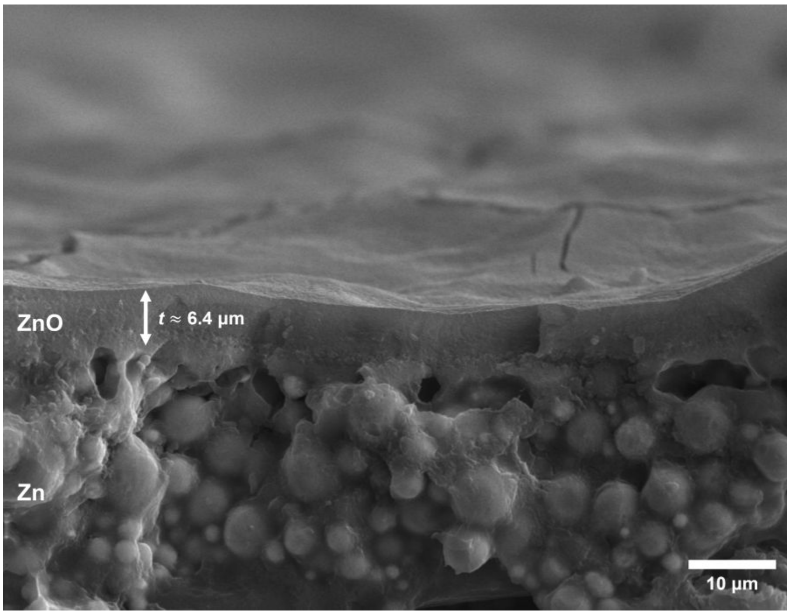

Figure S2. Cross-sectional SEM image of the Zn/ZnO interface, evidencing the ZnO film thickness.

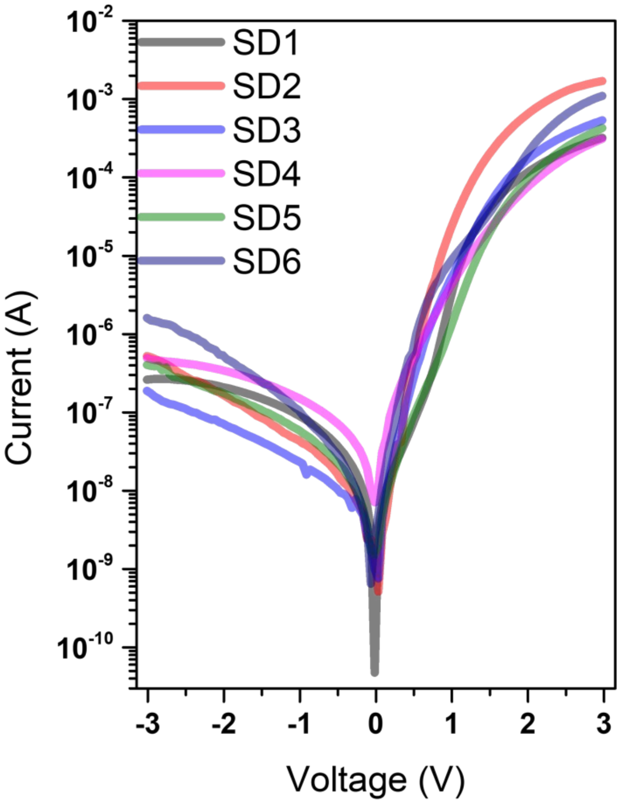

Figure S3. *I–V* characteristic curves in semi-logarithmic scale for six different SD samples in Zn/ZnO-NP/CNT architecture.

Table S1. All parameters calculated through Cheung's and Norde's method for the six SD samples in this study.

SD	RR	V_{on} (V)	Method	n	ϕ_b (eV)	R_s (kΩ)
1	1.2×10^3	1.43	Cheung	6.6	0.77	4.4 and 4.4
			Norde	-	0.77	9.06
2	3.3×10^3	1.37	Cheung	8.8	0.68	0.54 and 0.54
			Norde	-	0.69	0.05
3	2.9×10^3	1.56	Cheung	7.6	0.75	1.93 and 1.92
			Norde	-	0.75	2.52
4	0.64×10^3	1.84	Cheung	10.5	0.72	2.55 and 2.81
			Norde	-	0.72	1.71
5	1.1×10^3	1.9	Cheung	7.05	0.79	3.2 and 3.23
			Norde	-	0.79	2.65
6	0.7×10^3	1.87	Cheung	7.4	0.78	0.67 and 0.68
			Norde	-	0.73	4.65
$\bar{x} \pm \sigma$	$(1.6 \pm 1.2) \times 10^3$	1.7 ± 0.2	Cheung	8.0 ± 1.4	0.75 ± 0.04	2.2 ± 1.5 and 2.3 ± 1.5
			Norde	-	0.74 ± 0.04	3.3 ± 3.2

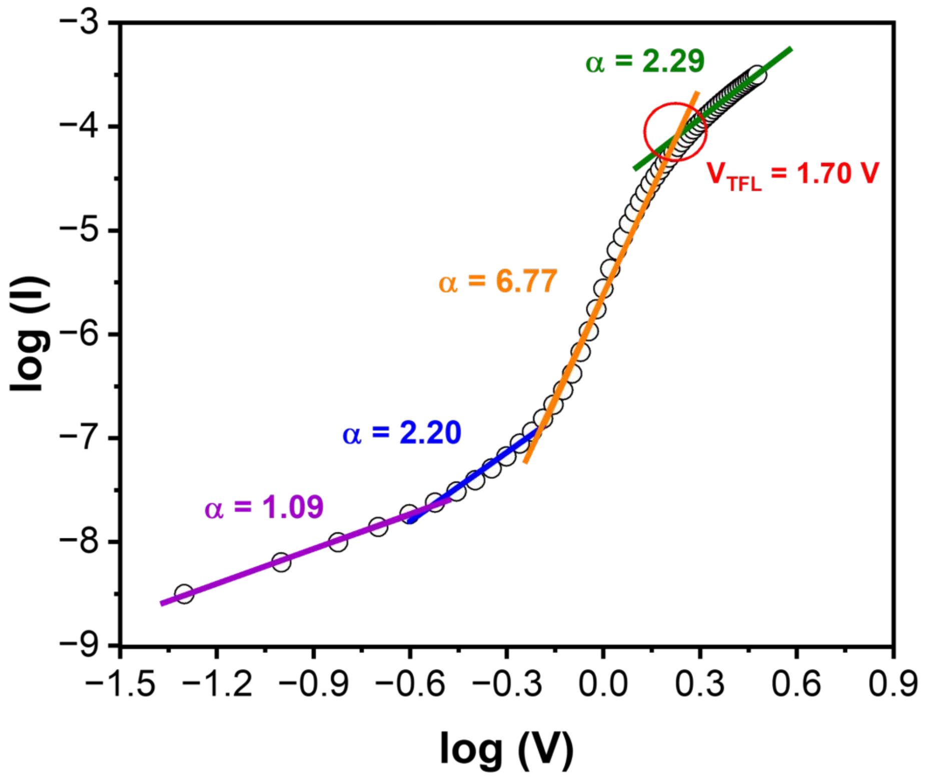

Figure S4. log–log plot of the I–V current versus voltage characteristics of the Zn/ZnO/CNT SD. Linear fits in different voltage regions yield the α slopes, and V_{TFL} is highlighted in red.
